# Tailored Multi‐Color Dispersive Wave Formation in Quasi‐Phase‐Matched Exposed Core Fibers

**DOI:** 10.1002/advs.202103864

**Published:** 2022-01-17

**Authors:** Tilman A. K. Lühder, Mario Chemnitz, Henrik Schneidewind, Erik P. Schartner, Heike Ebendorff‐Heidepriem, Markus A. Schmidt

**Affiliations:** ^1^ Leibniz Institute of Photonic Technology Albert‐Einstein‐Str. 9 Jena 07745 Germany; ^2^ Institut National de la Recherche Scientifique Centre Énergie Matériaux Télécommunications 1650 Boulevard Lionel‐Boulet Varennes Quebec J3X 1S2 Canada; ^3^ School of Physical Sciences and ARC Centre of Excellence for Nanoscale BioPhotonics (CNBP) and Institute for Photonics and Advanced Sensing (IPAS) The University of Adelaide Adelaide SA 5005 Australia; ^4^ Otto Schott Institute of Material Research Fraunhoferstr. 6 Jena 07743 Germany; ^5^ Abbe School of Photonics and Physics Faculty Friedrich Schiller University Jena 07743 Germany

**Keywords:** dispersive wave, quasi phase‐matching, soliton, tantalum pentoxide

## Abstract

Widely wavelength‐tunable femtosecond light sources in a compact, robust footprint play a central role in many prolific research fields and technologies, including medical diagnostics, biophotonics, and metrology. Fiber lasers are on the verge in the development of such sources, yet widespan spectral tunability of femtosecond pulses remains a pivotal challenge. Dispersive wave generation, also known as Cherenkov radiation, offers untapped potentials to serve these demands. In this work, the concept of quasi‐phase matching for multi‐order dispersive wave formation with record‐high spectral fidelity and femtosecond durations is exploited in selected, partially conventionally unreachable spectral regions. Versatile patterned sputtering is utilized to realize height‐modulated high‐index nano‐films on exposed fiber cores to alter fiber dispersion to an unprecedented degree through spatially localized, induced resonances. Nonlinear optical experiments and simulations, as well as phase‐mismatching considerations based on an effective dispersion, confirm the conversion process and reveal unique emission features, such as almost power‐independent wavelength stability and femtosecond duration. This resonance‐empowered approach is applicable to both fiber and on‐chip photonic systems and paves the way to instrumentalize dispersive wave generation as a unique tool for efficient, coherent femtosecond multi‐frequency conversion for applications in areas such as bioanalytics, life science, quantum technology, or metrology.

## Graphical abstract

Converting femtosecond radiation to multiple, user‐defined frequencies is of pivotal importance for many fields. Here, the authors present a breakthrough in realizing such a multi‐color source by generating multiple narrowband dispersive waves at predefined frequencies through quasi‐phase matching using a sophisticated optical fiber. Their method uses accurate local dispersion control via waveguide resonances in modulated nano‐films on exposed fiber cores.

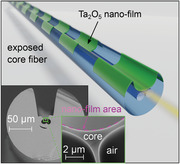

## 1 Introduction

Multicolor fiber light sources are of central importance for high‐end applications in medical diagnostics,^[^ ^1^ ^]^ biophotonics,^[^ ^2^ ^]^ and environmental science^[^ ^3^ ^]^ to reduce space requirements and system complexity and to open up novel fields of application. In particular, coherent femtosecond pulses at multiple wavelengths are particularly hard to realize in an environmentally robust, compact, single‐mode fiber format, yet promise unprecedented advances in nonlinear hyperspectral microscopy at low photo‐toxicity levels.^[^ ^4^, ^5^ ^]^ This need goes beyond the capabilities of commercially available supercontinuum sources, which usually emit broadband spectra and therefore do not have sufficient spectral power density or stability in the wavelength bands of interest.^[^ ^6^, ^7^ ^]^ Hence, current technical advances focus on utilizing parametric nonlinear optical processes (i.e., four‐wave mixing) to generate coherent light with high brightness in user‐defined spectral bands.^[^ ^8^, ^9^ ^]^ Yet, most approaches are bound to dual‐ or triple‐color outputs and picosecond pump pulses, inhibiting further advances, for example, in studying hyperspectral, femtosecond temporal dynamics in bio‐matter. As a consequence, novel approaches for generating coherent femtosecond light with high spectral intensities, preferably in multiple bands, are required.

Dispersive wave (DW) formation is a well‐established nonlinear frequency conversion scheme that would address those requirements, having found application in many fields such as spectroscopy,^[^ ^10^ ^]^ metrology,^[^ ^11^ ^]^ and medicine.^[^ ^12^ ^]^ From the physics perspective, DW generation is based on the creation of optical solitons, that is, femtosecond non‐dispersive optical pulses, and the phase‐matched emission of excess energy to linear radiation, i.e., DWs. The efficiency of this generation process allows the generation of notable intensities within just millimeters of propagation, which in turn allows maintaining femtosecond pulse durations for the DWs at the point of their generation. Hence, due to strong light–matter interaction and unique dispersion tuning capabilities, DW generation is particularly effective in optical fibers, having led to multi‐octave spanning outputs in the infrared^[^ ^13^, ^14^, ^15^ ^]^ or tunable light generation from the visible to the UV.^[^ ^16^, ^17^ ^]^

Even with the successful use of this scheme, unconventional concepts are required to further tailor DW generation to establish ultrafast nonlinear frequency conversion into user‐defined spectral bands. Promising approaches use gas‐ or liquid‐filled hybrid material fibers^[^ ^18^, ^19^ ^]^ to tune the waveguide dispersion by pressure or temperature which comes at the relatively high expense of system complexity and missing fiber system interconnectability. Here, quasi phase‐matching (QPM) represents another promising approach. Traditionally, QPM is known as periodic modulation of the underlying nonlinear polarizability aiming to suppress undesired back conversion in higher harmonic frequency generation—a scheme that has led to a multitude of scientific advances in quantum and extreme‐UV light sources (e.g., refs. ^[^ ^20^, ^21^, ^22^ ^]^). Just recently, QPM has been introduced as a consequence of a longitudinally varying waveguide dispersion, leading to a variety of novel nonlinear features in optical waveguides.^[^ ^23^, ^24^, ^25^, ^26^ ^]^ Indeed, the scheme was experimentally employed to adapt supercontinuum generation^[^ ^27^, ^28^ ^]^ and four‐wave mixing^[^ ^29^, ^30^ ^]^ in on‐chip waveguides, and modulation instability in diameter oscillating fibers.^[^ ^31^ ^]^ The effect is similar to Kelly sideband formation in amplifier chains.^[^ ^32^ ^]^ Conforti et al. introduced QPM for soliton‐pumped DW generation in hundreds of meter long dispersion‐varying fibers.^[^ ^33^ ^]^ Yet, the design and fabrication restrictions of such fibers limit the spectral extent of the DWs and lead to significant temporal broadening following their generation due to meter‐long period lengths. Hence, none of the earlier implementations could reveal the full potential of this scheme, namely femtosecond light generation at multiple, user‐defined frequencies.

In this work, we show generation of multiple well‐controlled and user‐defined spectral bands through tailored QPM to femtosecond higher‐order DWs over centimeter distances. The technique allows for simultaneous energy transfer into multiple regimes that cannot be reached with classical DW generation. This versatile generation process could be unlocked by extending our recently developed dispersion modulation scheme^[^ ^24^, ^34^ ^]^ that applies high refractive index nano‐films on the exposed side of a suspended‐core fiber.^[^ ^35^, ^36^, ^37^, ^38^ ^]^ Here, we mastered the process to obtain periodic longitudinal patterns to achieve for the first time wavelength‐tailored QPM for the efficient higher‐order DW generation in fibers with femtosecond pump pulses, uniquely leading to distinct narrowband spectral features with sub‐picosecond durations as suggested by realistic nonlinear pulse propagation simulations. Experiments with periodically dispersion‐modulated samples, nonlinear pulse propagation simulations, and phase‐mismatching considerations illustrate the nonlinear frequency conversion process, overall affirming the generation of additional peaks outside the spectral region associated with unmodulated samples. Therefore, our study fully unlocks the potential of ultrafast QPM‐DW generation for both basic research and applications.

## 2 Concept

Soliton‐based DW formation is a phase‐matched process involving the wave vectors of the different spectral features, suggesting that this process can generally be influenced by external geometric lattice vectors (Supporting Information of ref. [33]) which is commonly used in parametric oscillations such as second,^[^ ^39^ ^]^ third,^[^ ^40^ ^]^ and high harmonic generation.^[^ ^41^ ^]^ Within the context of soliton fission, the PM equation from a soliton at wavelength λ_*s*_ including a QPM term, transformed to an effective index representation (corrected version of ref. [27]), is given by

(1) $$n_{\mathrm{eff}}\left(\lambda_{s}\right)+\left(\lambda -\lambda_{s}\right)\frac{dn_{\mathrm{eff}}}{d\lambda }\left(\lambda_{s}\right)+\frac{\gamma P\lambda }{4\pi }=n_{\mathrm{eff}}\left(\lambda \right)+\frac{q\lambda }{\Lambda }$$

where *P* is the soliton power, γ is the nonlinear parameter, *n*_eff_ is the effective index of the mode, *q* denotes an integer value, λ is the wavelength, and Λ represents the period length (pitch). The left side of the equation corresponds to the soliton index, while the right side refers to the DW index including the QPM term.

**Figure 1:**
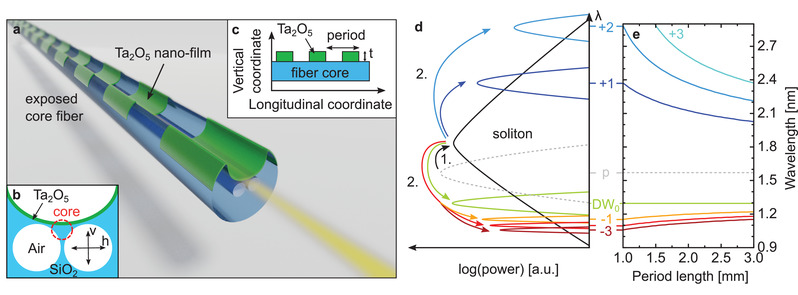
The concept of quasi‐phase‐matched dispersive wave formation in exposed core fibers that include periodically modulated nanofilms. a) Illustration of the exposed core fiber coated with a height‐modulated nanofilm with a schematic of the transversal and longitudinal cross‐sections shown in (b) and (c). d) Schematic of the QPM energy conversion process (1. Energy transfer from the pump to the fundamental soliton. 2. Energy transfer to the DW_0_ and QPM peaks of order ‐3 < m < +3). e) Dependency of the spectral position of the QPM‐related features shown in (d) on the period length.

Here, we establish a periodic effective index modulation through modulated waveguide resonances: Generally, placing elements with high refractive index adjacent to a waveguide core imposes optical resonances in the system in the form of new modes inside the element, which can lead to a substantial change of the waveguide dispersion.^[^ ^16^ ^]^ As shown, for example, in ref. [34], the inclusion of nano‐films enables waveguide systems to be used for nonlinear frequency conversion (through the creation of a zero‐dispersion wavelength [ZDW] near the pump), which typically do not work for this application. To establish the periodic modulation within the context of this work, we realize a periodic nano‐film on the core of an exposed core fiber (ECF) (**Figure** **1**a–c). The alternating sequence of sections with and without nanofilms leads to a periodically varying dispersion along the waveguide (pitch Λ, providing an additional grating vector 2π Λ^−1^ and thus yielding additionally PM opportunities for soliton‐based DW generation as suggested by Equation (1)). QPM‐DW peaks with negative *q* values are located on the short wavelength side of the *q* = 0 DW (referred in the following as zero‐order DW (DW_0_)) while the ones related to positive *q*‐values can be found on the long wavelength side of the soliton (Figure 1d). Note that smaller Λ increase the grating vector and thus every non‐zero *q* QPM‐DW is shifted further away from the DW_0_, whereas increasing the period length creates more phase‐matching wavelengths that are closer to each other (Figure 1e).

## 3 Results

**Figure 2:**
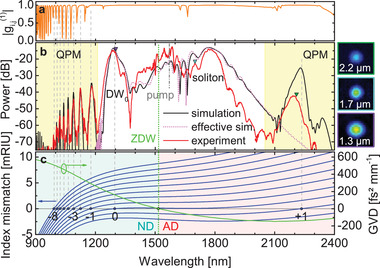
Measured output spectrum for a sample with a nanofilm period of 1.4 mm and a thickness of 74.4 nm in comparison to simulations. a) Ensemble coherence of 20 simulated spectra (noise is defined in the main text). b) Experimental (red) and simulated (black, using the realistic alternating dispersion) output spectrum at maximum input power of 192 mW before the lens. The pink dotted line represents a simulation of the spectrum utilizing the longitudinally invariant effective dispersion calculated with Equation (2). The yellow regions highlight the extended spectral regions addressed by the QPM process. The central pump wavelength is represented by the vertical grey dash‐dotted line. On the right are measured mode images at three different wavelengths corresponding to DW_0_, soliton and first QPM peak (color of frames match to triangles in the plot). c) Left ordinate: Spectral distribution of phase mismatch for different values of q, calculated by Equation (1). The phase matching wavelengths are marked with blue circles and dashed lines. Right ordinate: effective GVD (calculated with Equation (2)) with the ZDW marked with a green diamond and a dotted line, dividing this spectral interval in a normal dispersive (ND, light turquoise background) and anomalous dispersive domain (AD, light red background).

The measured spectrum of a nanofilm enhanced ECF with a pitch of Λ = (1.409 ± 0.005) mm and fiber length of 39 mm is presented in **Figure** **2**b. It is obtained by launching laser pulses with a width of 35 fs (FWHM) and a central pump wavelength of λ_*p*_ = 1570 nm at the maximum possible averaged input power of 192 mW into the fiber as depicted in the experimental setup (Figure S1, Supporting Information). Multiple QPM‐related peaks both on the short wavelength side of the DW and on the long wavelength side of the soliton are visible. Mode pictures with different filters prove fundamental mode operation at the DW, soliton, and QPM wavelengths.

To understand the different features of the spectral distribution, we first performed simulations assuming a longitudinally invariant dispersion. Both constant and alternating thickness nano‐film simulation contain a feature located at around λ = 1.3 µm which is related to the DW_0_. Note that the corresponding modulated nano‐film thickness needed to make the DW_0_ wavelength match the one of the constant thickness case is significantly larger. This indicates that the DW_0_ is created on the basis of a dispersion landscape influenced by both coated and bare fiber sections which is referred to in the following as “effective dispersion” (*gvd*_eff_). Comparing simulations with and without periodic modulation, this dispersion is given by the mean value of the effective refractive index of the fiber with (*n*_eff, w_) and without (*n*_eff, w/o_) nano‐film:^[^ ^31^, ^42^ ^]^

(2) $$\begin{array}{c} \begin{array}{c} \mathrm{gv}d_{\mathrm{eff}}=\frac{\lambda^{3}}{2\pi c^{2}}\frac{d^{2}\left[n_{\mathrm{eff},w}\mathrm{ff}+n_{\mathrm{eff},w/o}\left(1-\mathrm{ff}\right)\right]}{d\lambda^{2}}=\mathrm{gv}d_{w}\mathrm{ff}+\mathrm{gv}d_{w/o}\left(1-\mathrm{ff}\right) \end{array} \end{array}$$

Here, *c* symbolizes the speed of light and *ff* is the filling fraction with *l*_*nf*_ being the length of the nano‐film within one period (*ff* = *l*_*nf*_Λ^−1^). Details and graphical presentation of Equation (2) can be found in Figure S6, Supporting Information. Interestingly, *gvd*_eff_ does not depend on Λ for fixed *ff* and thus the effect of different period lengths on the QPM peaks can be easily investigated using the same effective dispersion. Moreover, reducing Λ to very low values to shift all QPM peaks out of the spectral region with significant QPM conversion efficiency can lead to interesting physics solely featuring the new effective dispersion which cannot be reached by the individual materials alone. Using this effective dispersion in simulations, the resulting spectrum (dotted line in Figure 2b) is similar to the spectrum without the QPM peaks in the wavelength range from the DW_0_ to the soliton, confirming the validity of Equation (2). In addition, this effective dispersion in combination with Equation (1) can accurately confirm the location of both QPM peaks with negative and positive *q* as well as the DW_0_ (Figure 2c). The mean error of the prediction for all peaks of the orders from ‐8 to 0 compared to simulations is 2 nm.

Because of the low input energy, modulation instability and the formation of high order solitons is avoided. As both these effects are associated with loss of coherence,^[^ ^43^ ^]^ this keeps our spectra deterministic and yield a high pulse‐to‐pulse stability. Calculating the ensemble first degree of coherence $|g_{i,j}^{\left(1\right)}|$ as in ref. [44] results in values close to 1 (Figure 2a), confirming excellent coherence even for the QPM peaks.

**Figure 3:**
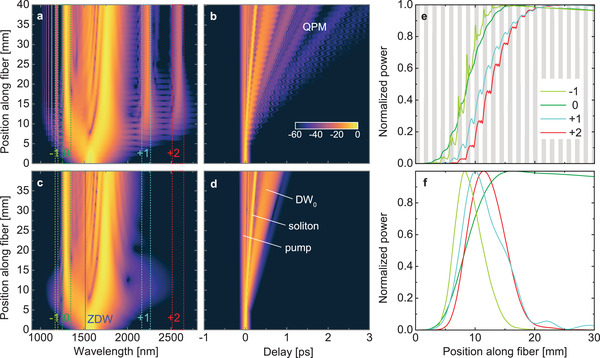
Simulated nonlinear pulse evolution for the periodic nanofilm‐enhanced ECF (period 1.4 mm, top row) in comparison to a fiber with corresponding effective constant dispersion (bottom row). a,c) Spatiospectral and b,d) spatiotemporal evolution of the simulated spectra (in dB) shown in Figure 2. e,f) Integrated normalized linear power evolution of the DW_0_ and QPM peaks marked and color‐matched in (a) and (c). In (e), the gray areas refer to the thick nano‐film regions. Please note that the curves in (e) and (f) are individually normalized and should not be compared in intensity.

The spectral and temporal pulse evolution of i) the real fiber that includes the periodic modulation and ii) the corresponding effective dispersion can be seen in **Figure** **3**a– d. Note that for the height‐modulated fiber, the reference time is correlated to the local group velocity at the pump wavelength, causing the “wiggle” type temporal evolution visible in Figure 3b. As in the final spectrum (Figure 2b), the pulse evolution assuming the effective dispersion matches the key features of the QPM‐related simulations that only exclude the QPM peaks. Specifically, the evolution in the effective dispersion case shows a distinct broadening of the spectrum at the soliton creation position about 5–20 mm from the fiber input. This is usually understood as a periodically occurring localized phenomenon of soliton self compression (SSC) also visible in other common soliton fission simulations.^[^ ^44^ ^]^ Note that although numerical calculations using the effective dispersion estimate the soliton number to be 3.4, no soliton fission to multiple fundamental solitons can be observed in the temporal simulations (Figure 3d). Comparing effective dispersion and periodically coated fiber simulations, especially considering the power evolution of distinct QPM peaks displayed in Figure 3e+f, the origin of the QPM peaks can be explained as follows:

Although DWs can grow from noise,^[^ ^44^ ^]^ the conversion efficiency increases in case they are seeded and amplified,^[^ ^45^, ^46^ ^]^ especially for lower pump power. That is why the SSC broadening provided here is crucial to permanently transfer power to the QPM features. This spectral overlap restricts the longitudinal domain of QPM energy transfer (called in the following as amplification domain) to the range 5 mm < z < 20 mm.

At the beginning of the amplification domain, the starting position (z‐position) of QPM peaks and the spectral broadening for the effective dispersion case coincide. The end of the amplification domain can be influenced by two different mechanisms: First, as in Figure 3, the end position of the QPM peak amplification matches the soliton broadening, that is, the *q* = +2 peak starts later compared to the *q* = +1 peak and both of them reach their maximum power later than the *q* = ‐1 peak. Second, temporal overlap of the soliton and the respective QPM feature also needs to be considered (similar to sum frequency processes^[^ ^47^ ^]^). In some cases at high input power where the soliton broadening is within a short fiber section only, the amplification domain is increased beyond the pure overlap area of the soliton broadening and the QPM feature. After the amplification domain, the power in each QPM‐peak slowly decreases following the linear losses included in simulations. The spectral extension of the SSC related broadening changes in total covered bandwidth and spatial extension along the fiber depending on input power and nano‐film thickness.

A detailed inspection of the signal evolution shows that QPM‐DWs with positive *q* grow within the coated sections of the fiber (grey regions of Figure 3e) and stagnate/decrease for the uncoated parts. Contrarily, for the negative *q* peaks, the power increases sharply near the transition region toward the start of coated regions. This difference of the creation points can also be seen in frequency resolved optical gating traces (see Figure S7 and movie, Supporting Information) and might be caused by the synchronous delay of soliton and positive *q* QPM peaks relative to the pump while negative *q* QPM peaks and the soliton exhibit counter‐propagating temporal movements.

In comparison to the QPM peaks, the DW_0_ is generated similarly in both the effective dispersion and the alternating dispersion case (dark green line in Figure 3e,f). Albeit somewhat distorted, it is amplified along both nanofilm enhanced and bare fiber regions. It is expected that the DW_0_ creation also requires spectral overlap with the soliton, which cannot be evidently seen in pulse propagation simulations. Only by switching off the QPM peaks using the effective dispersion, a clear spectral comparison for the QPM generation with and without additional spectral features can be made, strengthening the seeding hypothesis.

For different input powers, the QPM peaks stay almost at the same wavelength, whereas the DW_0_ and the soliton move away from the pump wavelength for increasing power. This is due to the steepness of the phase‐matching curves, which are flat around the DW_0_ and thus are spectrally more susceptible to small changes of the nonlinear phase and soliton wavelength (see Figure 2b). The power evolution is discussed in more detail in Figure S3, Supporting Information.

**Figure 4:**
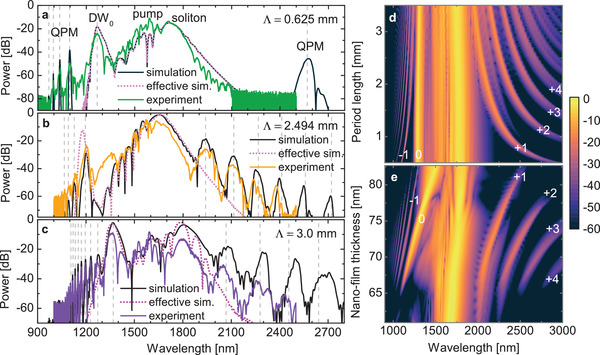
Simulated and experimental spectra at maximum input power (≈ 192 mW) for fibers with nano‐film periods (0.625 mm (a), 2.5 mm (b), 3.0 mm (c), details can be found in Table 1). Phase‐matching wavelengths calculated with Equation (1) are marked by dashed lines. Simulation of nano‐films with varying periods (d) and thicknesses (e) with otherwise constant parameters identical to the fiber of Figure 2 (peak power: 11.8 kW; nano‐film thickness: 74.4 nm; filling fraction: 52.1 %; fiber length: 40 mm). The color scale on the right is in units of dB.

QPM‐related DW generation has also been experimentally observed for other nano‐film periods. Here, **Figure** **4**a–c shows the experimental spectra at maximum input power for coated ECFs with different pitches, which are compared to corresponding simulated spectra, effective dispersion related spectra, and effective dispersion phase‐matching wavelengths. In accordance with Equation (1), greater periods lead to more QPM peaks located closer to each other, as simulated in Figure 4d with identical nanofilm thicknesses. The reason why the 3.0 mm sample has fewer peaks with positive *q* than the 2.5 mm sample lies in the nanofilm thickness. The maximum thickness contributes to the QPM location by influencing the effective dispersion and consequently shifting the DW_0_ as can be observed in simulations varying the nanofilm thickness (Figure 4e). Here, the *q* = +1 QPM peak moves almost parallel to the DW_0_. Interestingly, this peak can be created even at thin nanofilm thicknesses, where the conversion efficiencies to the DW_0_ and negative *q* QPM peaks practically vanish. There is a specific nanofilm thickness around *t* = 71 nm where the QPM peaks extend the most to the IR, which shifts toward thicker layers for longer period lengths. The experimental and simulation parameters of all samples are shown in **Table** **1**. Although the peak power in simulations is adapted to match experimental data and is different for each fiber individually despite similar experimental input power, it is to be expected that coupling efficiency varies between 10–20%.

**Table 1:** Experimentally measured, simulation matched, and calculated parameters for all presented ECF samples

Period^a)^	*ff* ^a)^	Fiber length^a)^	*P* ^b)^	*t* ^b)^	Loss^b)^	$\lambda_{s}$ ^b)^	$L_{D}$ ^c)^	$L_{\mathrm{NL}}$ ^c)^	$L_{f}$ ^c)^
[mm]		[mm]	[kW]	[nm]	[dB m^−1^]	[nm]	[mm]	[mm]	[mm]
0.625	0.588	34	8.5	70.8	10	1645	9.6	2.3	4.7
1.409	0.521	39	11.8	74.4	10	1675	18	1.6	5.3
2.494	0.511	36	7.7	68.8	10	1590	5.8	2.7	4.0
3.000	0.506	45	13.0	78.1	10	1690	106	1.4	12

*L*_D_, dispersive length; *L*_NL_, nonlinear length (as defined in ref. [65]); $L_{f}=\sqrt{L_{\mathrm{NL}}L_{D}}$, fission length; *ff*, filling fraction; *t*, nanofilm thickness.

a) Measured;

b) Simulation matched;

c) Calculated.

## 4 Discussion

The difference in the central regions of the power‐spectral evolution when comparing effective dispersion simulations to real fiber simulations is minor for small periods. The agreement reduces for greater periods, especially for the DW_0_, which can be explained as follows: Effective media usually include a characteristic length *L*_c_ that, when exceeded, breaks the effective approximation. For increasing periods (configuration of Figure 2 considered), the DW_0_ starts to red‐shift in simulations with alternating thickness starting from Λ ≈ 1.5 mm, although it remains constant in the effective simulation (according to Equation (2)). Here, *L*_c_ coincides with the nonlinear length, suggesting the formation of effective media on a macroscopic scale for the propagating pulse (details in Figure S6, Supporting Information). Note that the breakdown of the approximation does not influence QPM: Even for greater periods up to a few cm, clear QPM peaks emerge in simulations. For this effect, the number of periods within the amplification domain (Figure 3) is of importance.

The dispersion modulation via induced resonances results in a very high group velovity dispersion (GVD) modulation amplitude Δβ_2_. Within the context of optical fibers, we achieve here a record value of Δβ_2_ = 270 fs^2^ mm^−1^ at the pump wavelength, exceeding typical values achieved through core diameter in fibers variation by more than ten times. Note that this value is of the same order of magnitude as the values reported for planar high index contrast on‐chip waveguides (Table S2, Supporting Information), indicating that the very high GVD modulation amplitudes achieved in planar waveguide technology can be reached for the first time in fiber optics by our approach. This point is confirmed by preliminary modal simulations showing Δβ_2_ = 1300 fs^2^ mm^−1^ at λ = 1570 nm for a film thickness of 122 nm. Note that the sign of the GVD is inverted twice each period for λ < 1740 nm. However, this is actually not necessary: Even for small GVD modulation amplitudes (e.g., 64 nm < *t* < 64.8 nm (Figure S8, Supporting Information)), less intense QPM peaks at nearly the same wavelength are found (compare Figure 2, comparison of constant thicknesses in Figure S6, Supporting Information). Here, the GVD‐sign change occurs only within 1514 nm < λ < 1533 nm.

For shorter wavelengths, QPM peaks extend the spectrum down to λ = 1 µm. This does not increase the potential bandwidth, as this can also be achieved by shifting the DW_0_ using an appropriate constant nanofilm thickness.^[^ ^34^ ^]^ The situation changes, however, for the QPM peaks at long wavelengths: Here, the second ZDW at λ ≈ 2.7 µm is spectrally too far away to produce a zero‐order long‐wavelength DW by conventional means with our laser, so QPM is the only way to broaden the spectrum at low input energy.

A unique aspect of the ultrafast QPM‐DW scheme is the efficient energy conversion into multiple selected narrowband intervals when using a femtosecond laser source (35 fs FWHM, details in Supporting Information). Since there is no similar effect that can produce such high spectral densities at selected wavelength for femtosecond pump pulses, our concept defines an entirely new approach for tailored hyperspectral nonlinear light generation. Also worth mentioning is that, in contrast to classical DW generation, QPM enables the generation of spectral features in the anomalous dispersive range without relying on soliton‐self frequency shifts. Note that the peaks are extremely well‐defined in spectral location and bandwidth, underlining the high accuracy of the periodic modulation. Another advantage is the millimeter range of the modulation, which is technologically addressable straightforwardly and enables very good reproducibility.

To demonstrate the further potential of QPM‐DW generation, additional simulations have been conducted considering two hypothetical scenarios (details in Supporting Information). The first scenario assumes a 2 µm pump laser to involve the second ZDW at long wavelengths to generation process (Figure S10, Supporting Information). A filling of the spectral gap between DW_0_ and pump through multiple QPM‐DWs and light transfer up to λ = 4.5 µm is observed although silica losses are considered. The second scenario (Figure S11, Supporting Information) assumes no material losses (only wavelength‐independent 10 dB m^−1^ propagation losses) and a pump laser at 1.57 µm with substantially higher, yet feasible peak power. The results show efficient light transfer into multiple DWs far beyond 4.5 µm and suggest the beneficial employment of non‐silica fibers (e.g., InF_3_, chalcogenides, tellurites) to address the mid‐infrared wavelength domain. Remarkably, we observed a higher conversion efficiency of the QPM peaks compared to the DW_0_.

The origin of the effective dispersion lies in the combination of different modes and their corresponding effective indices. This new effective dispersion allows creating new dispersion landscapes that are not achievable in uniformly shaped fibers. Hence, resonance‐empowered dispersion management provides a general, versatile concept which could find application in highly sensitive dispersion management for precise spectral shaping^[^ ^24^, ^48^ ^]^ or dispersion flattened fibers^[^ ^49^, ^50^ ^]^ for triggering new advances in on‐chip nonlinear frequency conversion, supercontinuum generation, and optical signal processing.

One strategy to reduce the spectral distance between QPM peaks is to vary the period along the fiber. As a result, the conversion efficiency decreases compared to a fixed period as the amplification length for each local period length is reduced (compared to Figure 3). Simulations that demonstrate this effect by assuming a longitudinally varying period are shown in Figure S9, Supporting Information.

The range of period length of our QPM approach fills the gap between diameter‐modulated fibers with meter‐scale periods (ref. [33], [5 m], ref. [31] [10 m]) and structured waveguides with micrometer‐scale period lengths (ref. [27], [6 µm and 200 µm]; ref. [51] [7–29 µm]). Note that our periods are much larger than those used in parametric oscillation schemes and are technically easier to realize using our novel implementation approach, as shown in this work.

Ultrafast QPM‐DW generation produces a series of precisely localized, well‐controlled and power‐independent spectral bands using a small‐scale fiber device (e.g., Figure S3, Supporting Information). An unique advantage is the efficient energy transfer to specific bands at very low input powers yielding exceptionally high pulse‐to‐pulse correlation due to the low soliton numbers (Figure 2a). This is essential for many single‐pulse related applications, examples of which include discrete Fourier transformation (DFT)^[^ ^52^ ^]^ (e.g., real‐time analysis of chemical reactions or protein dynamics^[^ ^52^ ^]^ or sensing of environmental gasses^[^ ^3^ ^]^), metrology (e.g., frequency combs^[^ ^53^ ^]^), optical coherence tomography,^[^ ^54^ ^]^ and cascaded parametric effects in general. Yet, first and foremost, ultrafast nonlinear fiber light sources with tailored spectral properties and high coherence have the unique potential to unlock new applications within biophotonics as described in the work of H. Tu and S. A. Boppart.^[^ ^55^ ^]^ In this context, the characteristic multi‐peak output of our source allows to envision the realization of a so‐far unexplored type of ultrafast imaging source—i.e., a single‐excitation multi‐wavelength femtosecond laser for example, hyperspectral multi‐photon fluorescence imaging. In addition to applications as a multi‐color light source for multimodal imaging,^[^ ^1^ ^]^ a new class of hyperspectral phase‐sensitive sensors or microscopes can be envisioned which is based on the locked relative phases between the generated DWs: Here, one (or more) spectral band can experience a phase change imposed by a sample, which can eventually be referenced to one of the other non‐modulated QPM‐bands. In light of these unique perspectives, QPM‐DW generation may provide a key tool to approach those scientific and technological advances in the near future.

## 5 Conclusion 

Multi‐color fiber light sources are of central importance within many emerging areas such as life science, multimodal imaging, or metrology. Here, we show the generation of multiple well‐controlled and user‐defined bands through tailored ultrafast QPM to sub‐picosecond higher‐order DWs over centimeter distances, allowing to simultaneously transfer light into multiple regimes that cannot be reached with classical DW generation. The key to this flexible light conversion is a periodically varying waveguide dispersion with record‐high modulation amplitude in the context of optical fibers, realized by height‐modulated nanofilms on the core of exposed core fibers through a novel fabrication approach. Based on experimental data, nonlinear pulse propagation simulations and QPM considerations, the observed physics is clearly associated with the generation of solitons and the generation of multi‐order DW, with excellent agreement between simulated data and measurements. Multiple phase‐matched DWs on both sides of the conventional spectrum have been found and can be tuned with nano‐film thickness and period length. These features extend the output, especially into the infrared region, beyond the capacities of a comparable unmodulated waveguide. Particularly, the QPM‐DW scheme broadens the spectrum beyond the limited soliton Raman shift, which is beneficial compared to meter‐long, single‐ZDW fibers. From the design perspective, the spectral positions of soliton and zero‐order dispersive wave are fully explained by an averaged effective index weighted by the filling fraction, leading to an effective GVD distribution. This gives the foundation for accurate, a priori prediction of the QPM wavelengths.

Through our detailed analysis, we have been able for the first time to unlock the practical potential of ultrafast QPM‐DW for both basic research and applications. A key feature is the efficient energy conversion into multiple selective narrowband spectral intervals with a femtosecond laser source (35 fs), a conversion which cannot be achieved by means of other approaches. In contrast to the zero‐order DW and soliton Raman shifting sources, QPM peaks offer almost input power independent wavelength‐stable multi‐peak frequency conversion to application‐relevant wavelengths at very high levels of coherence, opening up new pathways toward previously inaccessible parameters for new applications, including single‐excitation hyperspectral nonlinear imaging or ultrafast phase sensitive detection. As demonstrated by additional simulations, the presented light conversion scheme also offers a practically feasible potential for transferring light to distant spectral intervals, for example, in the mid‐infrared, or to fill up spectral gaps in the anomalous dispersion regime.

In addition to QPM, the realization of effective dispersion opens many degrees of freedom in the design of complex dispersion landscapes to optimize nonlinear frequency conversion processes (e.g., supercontinuum generation, parametric processes). The ultrafast QPM approach presented here is not limited to nanofilm‐modulated fibers and can be readily applied to other waveguide systems, including both optical fibers and on‐chip systems.

Overall, by harnessing the presented versatility and efficiency of the approach, the presented nonlinear frequency conversion scheme will find unique use in a multitude of spectroscopy and sensing related areas such as bioanalytics, life science, quantum technology, or metrology, particularly in situations demanding well‐controlled light in selected spectral bands with excellent pulse‐to‐pulse stability.

## 6 Experimental Section

### Sample Fabrication

ECFs were fabricated from fused silica glass using a technique described previously.^[^ ^56^ ^]^ Periodically varying nano‐films (Ta_2_O_5_) on ECFs were created by carefully aligning the fiber and its core in a reactive magnetron sputtering chamber and periodically shielding the waveguide core by a laser‐cut aluminium foil line lattice mask placed directly on top of the fiber (see Figure S2, Supporting Information). This deposition procedure resulted in alternating fiber sections with and without nanofilm along the entire fiber length (Figure 1c). The possible period lengths ranged from 120 µm up to several mm, limited to the short period side by the nano‐film edge sharpness, needing a transition length of ≈60 µm to reach 5–95 % thickness. Shorter period lengths require a mask closer to the core which, in this case, was restricted by the fiber geometry. Within the context of nonlinear broadening, useful thicknesses of the nanofilm reached up to 85 nm with 100 % modulation depth, forming a trapeze nanofilm shape with smoothed edges.

### Experimental Setup

The nonlinear frequency conversion process was analyzed by a nonlinear transmission setup: A pulsed ultrafast laser (pulse duration: 35 fs FWHM, repetition rate: 80 MHz, central pump wavelength: λ_*p*_ = 1570 nm, FFpro IRS II, Toptica Photonics) with a polarization parallel to the nano‐film (h‐polarization in Figure 1b) was launched into the nano‐film coated fiber with an aspheric lens (C230TME‐C, Thorlabs Inc.). The resulting broadened spectrum was captured and the spectrum was measured by an optical spectrum analyzer (AQ6375, Yokogawa Inc.). Details of the experimental setup and the color aberration correction can be found in Figure S1, Supporting Information.

### Simulations

The nonlinear pulse propagation was simulated by solving the generalized nonlinear Schrödinger equation based on code from Dudley and Taylor.^[^ ^57^ ^]^ Simulations included shock, a purely silica Raman response with a fraction of 0.18 (ref. [58]), correction for linear effective mode area dispersion,^[^ ^59^ ^]^ and full dispersion of the fiber (calculated using a finite element solver [Comsol Multiphysics] based on SEM images of the fiber, Figure S2d, Supporting Information). Constant averaged losses along the fiber were assumed and adapted to match experiments and simulations, resulting in 10 dB m^−1^. Material dispersions were taken from refs. [60] and [24] with the material nonlinearities of refs. [61, 62]. Having period lengths much longer than the transition length of the real nano‐film edge, the longitudinal nanofilm shape in simulations was simplified as rectangular with the coated regions of the fiber having a constant thickness. For coherence considerations, 20 individual simulations were performed including noise using a 10^12^‐fold multiplied one photon per mode model with random phase,^[^ ^63^ ^]^ forming a noise floor of ‐35 dB. On top of it, random pulse energy and pulse lengths variations of up to 2% were added, being realistic for fiber lasers with telecommunications wavelengths.^[^ ^64^ ^]^

## Conflict of Interest

The authors declare no conflict of interest.

## Disclosures

The authors declare no conflict of interest.

## Supporting information

Supporting Information

Supplemental Video 1

Supplemental Video 2

## Data Availability

The data that support the findings of this study are available from the corresponding author upon reasonable request.
