# Supplementary material for: Tailored Multi‐Color Dispersive Wave Formation in Quasi‐Phase‐Matched Exposed Core Fibers
Source: Adv Sci (Weinh). 2022 Jan 17;9(8):2103864. doi: 10.1002/advs.202103864 (PMC8922130; doi:10.1002/advs.202103864)
Supplement: Supplementary file 1 — Supporting Information [file ADVS-9-2103864-s002.pdf]

ADVANCED SCIENCE
Open Access

Supporting Information

for *Adv. Sci.*, DOI 10.1002/advs.202103864

Tailored Multi-Color Dispersive Wave Formation in Quasi-Phase-Matched Exposed Core Fibers

*Tilman A. K. Lühder, Mario Chemnitz, Henrik Schneidewind, Erik P. Schartner, Heike Ebendorff-Heidepriem and Markus A. Schmidt**

ADVANCED SCIENCE
Open Access

Supporting Information

for *Adv. Sci.,* DOI: 10.1002/advs.202103864

Tailored Multi-Color DispersiveWave Formation in Quasi-Phase-Matched Exposed Core Fibers

*Tilman A. K. Lühder, Mario Chemnitz, Henrik Schneidewind, Erik P. Schartner, Heike Ebendorff-Heidepriem, and Markus A. Schmidt**

Supplementary Information for:

Tailored Multi-Color Dispersive Wave Formation in Quasi-Phase-Matched Exposed Core Fibers

Tilman A. K. Lühder[1], Mario Chemnitz[2], Henrik Schneidewind[1], Erik P. Schartner[3], Heike Ebendorff-Heidepriem[3], and Markus A. Schmidt*[1,4,5]

[1] Leibniz Institute of Photonic Technology, Albert-Einstein-Str. 9, 07745 Jena, Germany
[2] Institut National de la Recherche Scientifique, Centre Énergie Matériaux Télécommunications, 1650 Boulevard Lionel-Boulet, Varennes, Quebec J3X 1S2, Canada
[3] School of Physical Sciences and ARC Centre of Excellence for Nanoscale BioPhotonics (CNBP) and Institute for Photonics and Advanced Sensing (IPAS), The University of Adelaide, Adelaide SA 5005, Australia
[4] Otto Schott Institute of Material Research, Fraunhoferstr. 6, 07743 Jena, Germany
[5] Abbe School of Photonics and Physics Faculty, Friedrich Schiller University, 07743 Jena, Germany
* markus-alexander.schmidt@uni-jena.de

Experimental setup

The experimental setup is depicted in Figure S1 and consists of the laser, an attenuator based on a rotating $\lambda/2$ plate combined with fixed polarizer, a second $\lambda/2$ plate to control the polarization and a C230TME-C aspheric incoupling lens (Thorlabs Inc.) in front of the sample. At the outcoupling side, the experimental configuration used depends on the actual spectral range addressed. For the NIR range, the spectrum is captured with an 20x NIR Objective (Mitutoyo GmbH) and a silica low-OH fiber with 400 nm core diameter for best color correction. For wavelengths larger than 1.8 µm, these elements are replaced by an aspheric lens C028TME-D (Thorlabs Inc.) and an InF_3 fiber (MF12L2, 100 µm core diameter, Thorlabs Inc.) for better transmission. Starting from 1.9 µm, a long pass filter 1650 nm (Edmund Optics) is inserted after the outcoupling lens to prevent measuring the second order of short wavelength peaks. Principally, an optical spectrum analyzer (AQ6375, Yokogawa Inc.) in experimental extended range mode (1000 nm $< \lambda <$ 2500 nm) is used to measure the spectra, while for $\lambda < 1.27$ µm in Figure 4a it was replaced by a model AQ6315A (Ando Corp.). All spectra consist of multiple measurements with the outcoupling optimized for different wavelengths that are stitched together, corrected for the offset of changing components by measuring at the same optimized wavelength. For mode image capturing, an IR camera (ABS GmbH, Jena) replaces the collection fiber. Spectral selection is done by filters (Figure 2): bandpass FB 1310-12 (Thorlabs Inc.), a combination of a long pass filter 1650 nm (Edmund Optics) with a metal interference filter IF 1700 nm (Carl Zeiss Jena GmbH), and a bandpass filter BP 2211-82 (Laser Components GmbH).

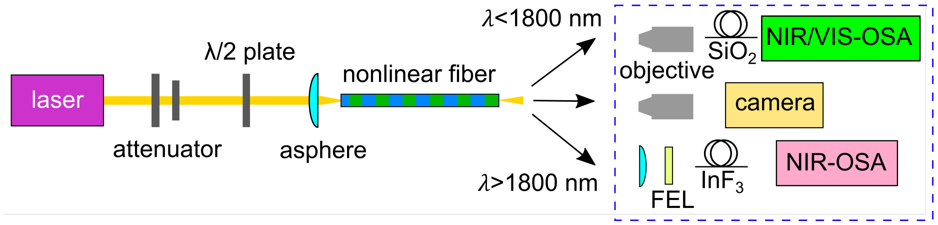

Figure S1: Optical Setup. The elements in the blue box indicate the various configurations of the diagnostics. The silica collection fiber and the NIR objective, designed up to $\lambda = 1.8$ µm, can be replaced by a D coated asphere and an InF_3 fiber for measuring longer wavelengths and a longpass filter (FEL) needs to be inserted. For mode images, a camera was inserted instead at the place of the collection fiber.

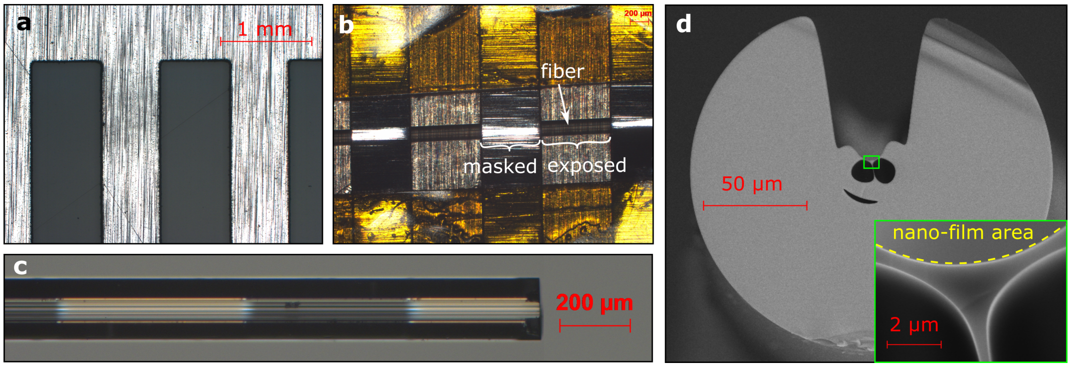

Figure S2: Preparation of the periodic height-modulated nano-film. (a) Aluminium foil mask prepared through cutting with a laser. (b) Aluminium mask on top of a fiber which is fixed with yellow Kapton tape ready for deposition. (c) Exposed core fiber coated with a periodic nano-film aligned to allow a view to the core. (d) SEM image of the fiber cross section with a magnified inset of the core region.

Sample preparation

Periodic nano-film coatings are produced by masking part of the fiber in a reactive magnetron deposition chamber. The mask was cut with a TruMark Station 1000 commercial laser cutter (Trumpf GmbH) and consists of standard supermarket aluminum foil. This offers great flexibility in the design of period length, filling fraction and allows theoretically for various profiles such as chirped gratings. In our case, rectangular holes with a height of 4 mm and a width of half a period were lined up to form a line lattice. With a microscope, the exact filling fraction including errors of the line width of the laser cut can be determined. The period lengths are measured with a vernier calliper over all periods. Fixing the mask on the pre-aligned fiber is accomplished by fixing it to the substrate with Kapton tape using the elongated holes (Figure S2b). This ensures the closest possible distance of the mask to the fiber to have sharp edges at the positions of thickness variation. The remaining transition region is visually estimated to be around 60 µm (Figure S2c) which is verified by a test using a spacer on a planar substrate resembling the fiber groove depth and a stylus profilometer. The shape of the nano-film profile does not influence the QPM peak position and even having sine shape films can produce odd harmonics, which is in accordance with the findings of Hickstein et al.[1]. For our simulations, the distribution of the nano-film is simplified to a sharp rectangular shape.

Tantal is sputtered in an 0.5 Pa argon atmosphere with 30 % oxygen content at 200 W dc. The amorphous nano-film thickness is expected to vary not more than 10 % within the used area. Small particles disturb the otherwise flat nano-film with a RMS of 0.2 nm and contribute to scattering losses. More details on the deposition profile and nano-film surface is published in Ref.[2].

Power dependency

Figure S3 shows the experimental and simulated input power dependency of the spectrum for the ECF with a period of 1.4 mm (the same sample as used within the context of Figure 2). Although starting with a transform limited sech^2 pulse instead of the deformed experimental one, the location of soliton, DW_0 and QPM peaks can be well recreated. This also holds for different input energies, as shown by the dashed lines in Figure S3 marking phase-matching wavelengths calculated with Eq. (1) using the soliton wavelengths also indicated by the dashed blue line in that figure. The used soliton wavelength is the one at its creation point before Raman shifting, and thus is not overlapping the one at the fiber end shown in the figure. The phase-matching curves confirm nearly power

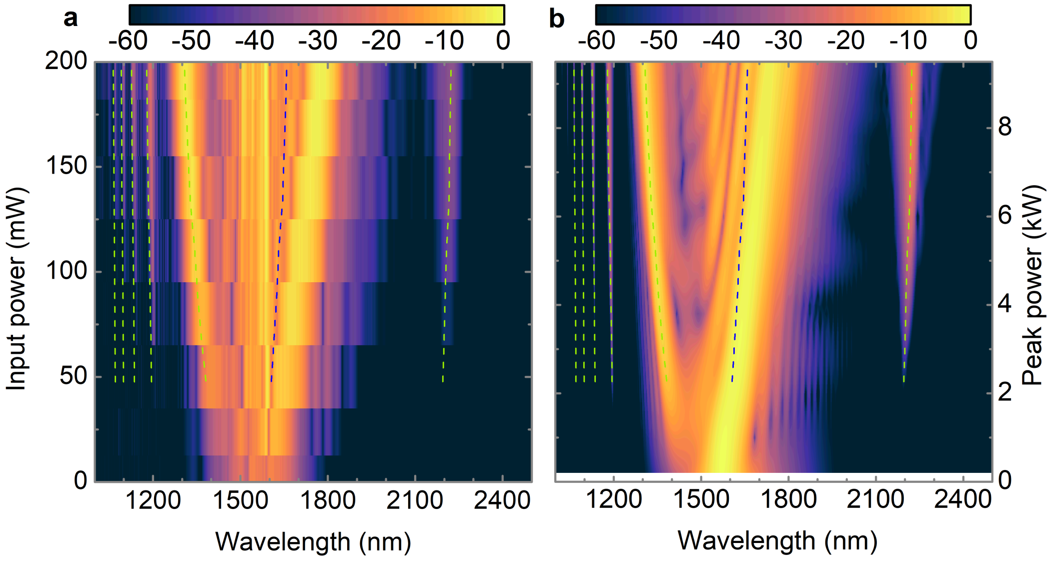

Figure S3: Power-spectral evolution. (a) Normalized experimental and (b) simulated input power dependent spectrum (in dB) for the ECF with a period of 1.409 mm, also used for Figure 2. The light green dashed lines represent phase-matching wavelengths using Equation (1) with the soliton wavelength marked in blue.

independent QPM peaks in contrast to the red-shifting soliton and blue shifting DW_0, which is a commonly observed effect. The reason is the very flat phase-mismatching curve near the zero crossing for the DW_0 compared to that of the QPM peaks as discussed in the main manuscript (see Figure 2c). The simulation with parameters matching the experiment of Figure 2b is compared to other experiments using the same fiber, but different incoupling conditions. Therefore, the calculated lines do not perfectly match the experimental spectra shown in Figure S3a.

Estimation of temporal widths of QPM-waves

The duration of the individual QPM-waves have been estimated by extracting the spectral bandwidth $\Delta\lambda$ at the maximum input power using the experimental data shown in Figure S3 of the Supplementary Information and calculating the bandwidth-limited pulse duration $\Delta\tau$, leading to the values presented in Table S1 (spectral resolution of optical spectrum analyzer: 2 nm). Although the DWs disperse in time, the fiber could be cleaved after the amplification domain close to the DW creation position at a fiber length of 20 mm in case of the example of Figure 3. The QPM peaks experience a walk-off of 280 fs, 140 fs, and 336 fs during their creation process for the DW of order -1, 0 and +1 compared to the soliton, respectively. These numbers in combination with FROG

Table S1: Estimation of temporal and spectral properties of the QPM-DW-waves (-3 dB).

dispersive wave order (DW_x)	DW spectral bandwidth $\Delta\lambda$ [nm]	bandwidth-limited pulse duration $\Delta\tau$ [fs]
-4	4.1	290
-3	3.9	320
-2	5.2	255
-1	11	136
0	32	54
1	43	119

simulations (Figure S7) suggest that the QPM-waves have durations of only a few hundred femtoseconds, which emphasizes the application potential of our light generation scheme for ultrafast applications such as pump-probe spectroscopy or hyperspectral imaging.

Losses

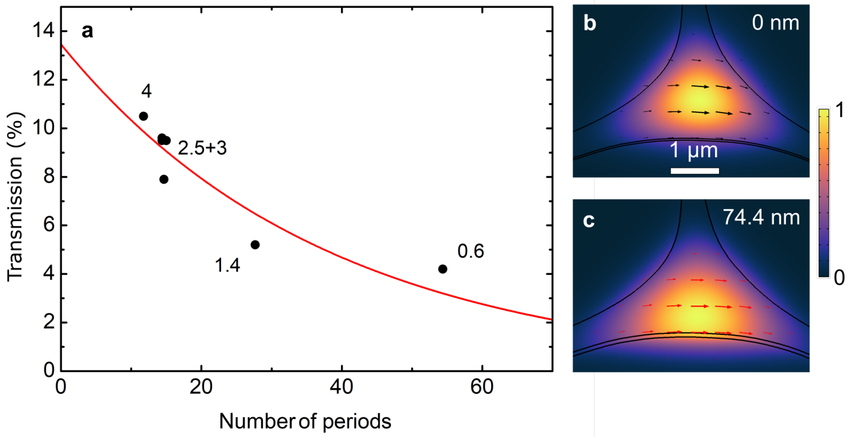

Figure S4: Transmission through periodic nano-film enhanced ECFs as a function of the number of periods. (a) Total transmission of all samples with different period lengths (marked in the figure) as a function of the total number of periods within the fiber length. The red line is an exponential fit to the data points. (b) Fundamental mode (norm electric field) without and (c) with 74.4 nm nano-film at $\lambda = 1.6$ µm.

In our simulations, loss is assumed to be constant and homogeneous across all wavelengths. This is a simplification, as in the real fiber material attenuation (e.g., OH groups in silica) and scattering losses (resulting from the field at the various interfaces) show characteristic spectral dependencies and strongly increase for wavelengths > 2.3 µm. This is the reason why the experimental spectra have lower power compared to simulations on the long wavelength side. Note that the open structure makes the fiber susceptible to pre- and post-coating contamination of the surface, which could be minimized by protecting it with, e.g., a heat shrink tubing. Moreover, losses are different for the coated and uncoated regions of the ECF, and we expect distinct losses at the transition region of the nano-film. As seen in Figure S4a the total transmission through the fibers (including lenses/objectives) is dependent on the number of periods of the entire fiber. Despite small differences in nano-film thickness, coupling efficiency, and fiber length, the transmission roughly follows the exponential Lambert-Beer law. This indicates that there is a constant loss when adapting the mode field within each period, as shown in Figure S4b+c for $\lambda = 1600$ nm. These modes of the nano-film and bare fiber sections have an overlap integral of their electric fields of 85 % suggesting the same amount of remaining transmission per half period. The experimental data, however, shows a substantially larger transmission of about 98.7 % per junction, obtained from exponentially fitting the data points of Figure S4a. The difference can be explained by a smoothing of the nano-film edge in experiments, exhibiting a 60 µm transition region, which leads to a more adiabatic transition showing less losses. On top of it the mode overlap integral is only calculated at one single wavelength.

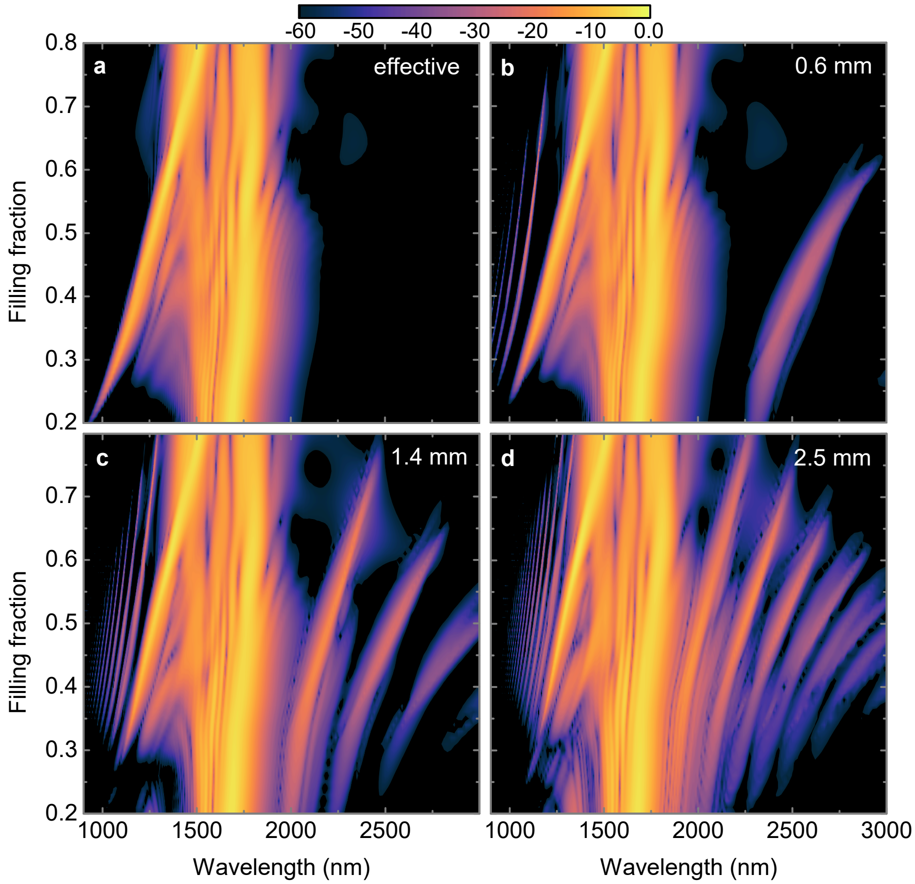

Figure S5: Spectral evolution as a function of filling fraction. Simulation of the influence of filling fraction on the spectrum at otherwise constant parameters (λ_p = 1570 nm, peak power: 10 kW, pulse duration 50 fs (FWHM), t = 74.4 nm). (a) effective dispersion, (b) 0.6 mm period, (c) 1.4 mm period, and (d) 2.5 mm period.

Filling fraction

Figure S5 shows the filling fraction dependent spectrum at maximum input power for the effective dispersion case (a) as well as for several period lengths ((b): 0.6 mm, (c): 1.4 mm, (d) 2.5 mm). All plots have the same effective dispersion, i.e., the central broadening domains should be identical, with only the QPM peaks experiencing changes. This statement is valid for most of the configurations, while there are visible differences, especially at small ff, related to short wavelengths and long period lengths. In both cases, the number of periods within the interaction region creating DW and QPM peaks is small. This is reasonable, as more periods lead to a more precise description of the effective medium approximation. The simulations also confirm the ff dependency of Equation (2), since the effective dispersion and alternating thickness simulations match for all other cases. Moreover, the simulations show great similarities with the nano-film thickness study of Figure 4e, verifying the nano-film shape independence since no conversion efficiency enhancement to particular q values for certain ff can be observed. Note that $ff \sim 0.5$ seems to provide the best results, compared to off-center ff values with adapted nano-film thickness.

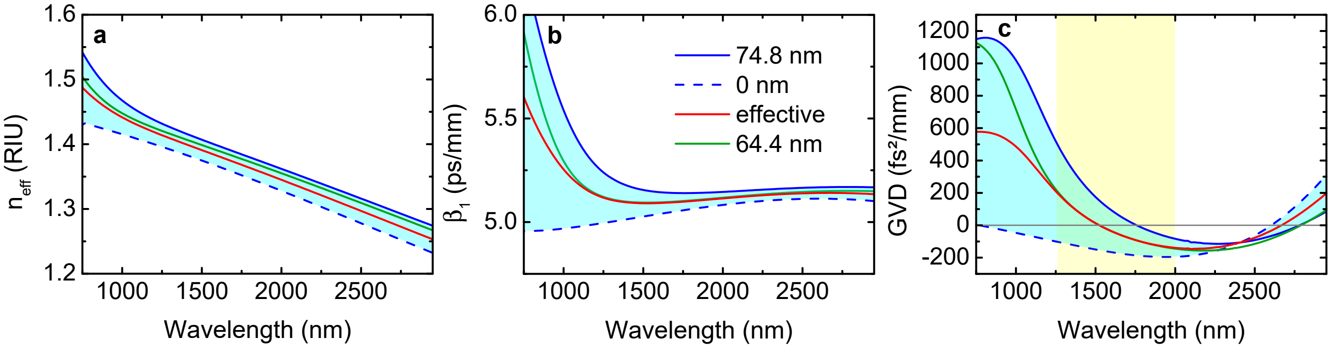

Figure S6: Effective dispersion. Comparison of various dispersion parameters for ECFs with nano-film thickness of t = 74.8 nm, t = 0 nm, t = 64.4 nm, and the mean of 0 nm and 74.8 nm (effective with filling fraction 0.5): (a) Effective refractive index neff, (b) β_1, and (c) group velocity dispersion. The cyan shaded area marks the *ff*-related tuning region for alternating layers with t = 0 nm and t = 74.8 nm. The yellow region highlights the matching region of GVD of effective and t = 64.4 nm dispersions.

Effective dispersion

The effective dispersion results from averaging the effective index over one period as described by Equation (2). It is beneficial to compare the t = 74.8 nm to t = 0 nm step lattice and its effective dispersion to the case of a constant intermediate nano-film thickness leading to similar spectra, here, being t = 64.4 nm thick. In Figure S6 the relevant curves are displayed, where the effective GVD (red line) matches well with the one with a t = 64.4 nm nano-film (green line) for 1250 nm $< \lambda <$ 2000 nm (yellow area). However, there are differences outside that central wavelength region where the effective index as well as β_1 do not match. By using these two dispersions (effective and constant 64.4 nm, see also the inset of Figure S8) in Equation (1) to match the wavelengths of the QPM peaks in the spectral region outside 1250 nm $< \lambda <$ 2000 nm unambiguously reveals that only the effective dispersion can explain the experimental results. Thus, we believe that the averaged GVD represents the correct underlying modal dispersion. Since the GVD inherits unknown integration constants when defining the entire fiber dispersion, the averaged effective index needs to be the physical origin of the effective dispersion. The difference of the effective GVD to its constant nano-film counterpart implies that by creating an effective dispersion, new dispersion landscapes are possible that are otherwise not realizable by constant nano-film modified ECFs. This is especially obvious when analyzing the tuning possibilities with different *ff* from 0 to 1, marked as a shaded area in Figure S6, which does not overlap with the constant 64.4 nm curve at all wavelengths.

FROG simulation

Frequency-resolved optical gating (FROG) simulations were calculated both for the fiber with a period of 1.4 mm and its effective dispersion counterpart. As an example, the pulse is displayed in Figure S7 at longitudinal position z = 19.5 mm, while the full evolution can be seen in the two Supplementary movies. Note that as the dispersion changes within each period, the relative delay of spectral components alternate, making the FROG simulations wiggling in the periodic nano-film situation.

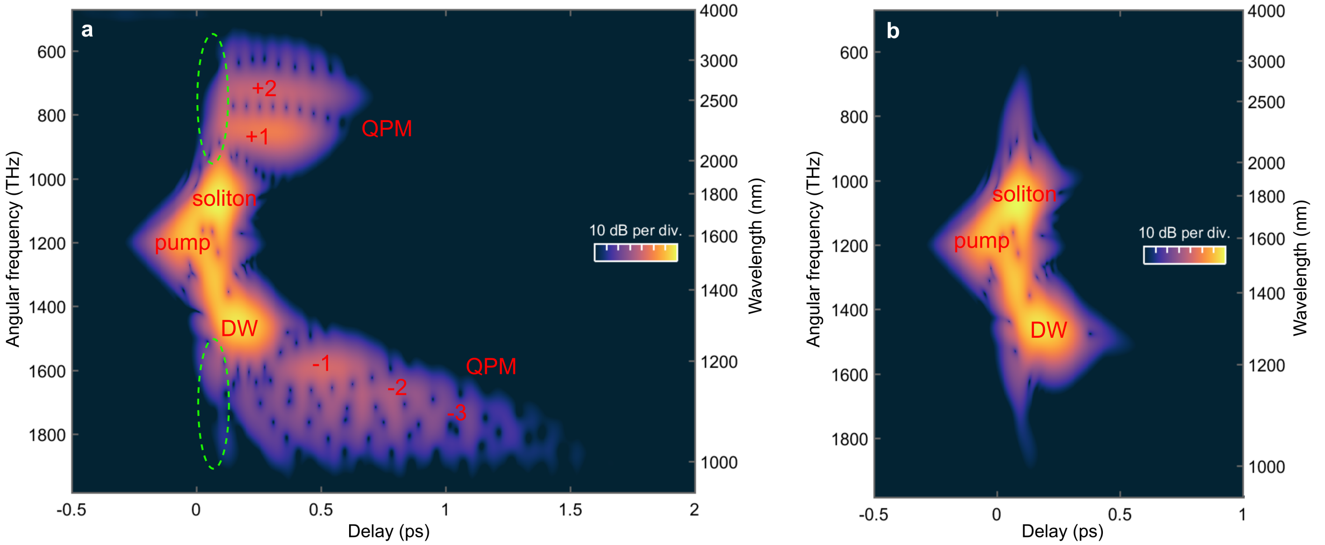

Figure S7: Frequency-resolved optical gating (FROG) simulation (λ_p = 1570 nm, peak power: 10 kW, pulse duration 50 fs, t = 74.4 nm, λ = 1.409 mm). FROG simulation with (a) alternating thickness and (b) effective dispersion at a fiber length of 19.5 mm (gating: 60 fs sech^2). QPM creation regions are marked with green ellipses.

Modulation depth

Figure S8 shows the enhanced conversion efficiency of the QPM process when using the full modulation depth of the nano-film in comparison to a reduced one with a similar effective dispersion. When analyzing the wavelengths of peaks outside the core spectrum (i.e., q = -3 and smaller, inset of Figure S8) the difference in effective dispersion can be acknowledged (compare to Figure S6).

A comparison to GVD modulation amplitudes used in other works can be found in Table S2.

Table S2: Comparison of the group velocity dispersion modulation amplitude of different types of periodically modulated fibers. For comparison, the parameters of three planar waveguides are also shown (in gray).

waveguide type	modulation method	GVD modulation amplitude at the pump wavelength	reference
fiber	mode anti-crossing	270 fs^2 mm^{-1}	this work (Figure 2)
fiber	outer fiber diameter	3 fs^2 mm^{-1}	Droques et al.[3]
fiber	outer fiber diameter	2 fs^2 mm^{-1}	Copie et al.[4]
fiber	outer fiber diameter	1.2 fs^2 mm^{-1}	Conforti et al.[5,6]
fiber	splicing of fiber sections	45 fs^2 mm^{-1}	Zia et al.[7]
on-chip	waveguide width	364 fs^2 mm^{-1}	Driscoll et al.[8]
on-chip	periodical poling	15 fs^2 mm^{-1}	Jankowski et al.[9]
on-chip	waveguide width	1000 fs^2 mm^{-1}	Lefevre et al.[10]

Longitudinally varying period length for QPM

One attempt to enhance the number of phase-matching opportunities relies on using a gradually increasing period, as also considered by Langrock et al.[11]. Here, this adaptive period length L_{p} depends on period number N_{p}, leading

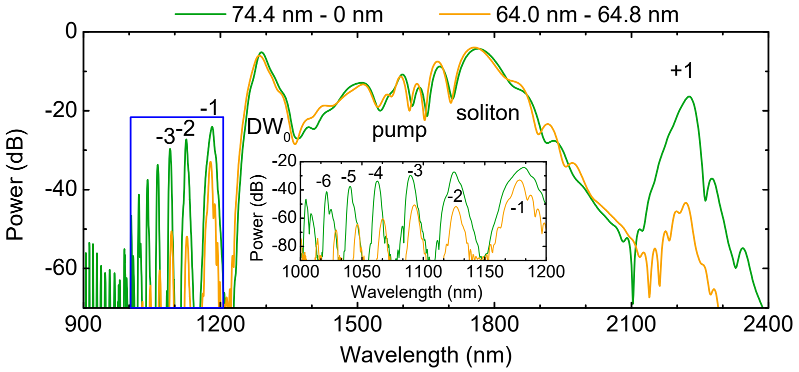

Figure S8: Modulation depth dependence (simulation). Comparison of the output spectra of the simulated fiber with a period of 1.4 mm (green) with a similar fiber with reduced nano-film modulation (orange) and otherwise identical parameters (λ_p = 1570 nm, peak power: 10 kW, pulse duration 50 fs, λ = 1.409 mm). The legend on the top shows the different thicknesses assumed, and the inset magnifies the dense QPM peaks with negative q-value (blue box).

to $L_\mathrm{p}(N_\mathrm{p})$ = 0.5 mm $\cdot(1{+}0.0006\ \cdot(N_\mathrm{p})^3)$. The region where the QPM peaks are created is from 5 to 20 mm along the fiber length (discussed in Figure 3), effectively limiting the usable gradient of this simulation to 0.7 mm $< \Lambda <$ 3 mm (Figure S9a). The maximum possible peak height of the first order DW (±1) is shown for a comparison in Figure S9b. Starting at a constant period length of 0.5 mm with QPM peaks far away from the soliton (near 3 µm), the conversion efficiency is low because the seeding power at those wavelengths is low. Increasing the period results in peaks closer to the pump, raising the conversion efficiency until it drops again because the total number of periods within the amplification region drops significantly. This is especially visible for the q = -1 peak at the DW_0 side.

Using a gradually changing period can help to increase the density of peaks in a spectral interval, as a variety of

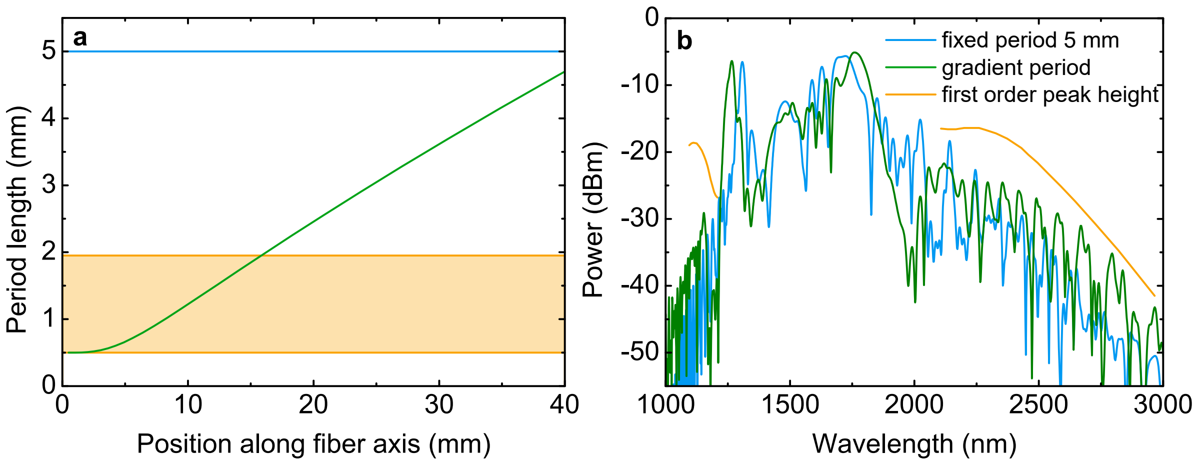

Figure S9: QPM-DW generation in case of a modulated period. (a) Nano-film configurations used in this simulation (pulse duration 50 fs, Peak power 10 kW, pump wavelength 1570 nm, nano-film thickness t = 85.5 nm, constant losses 20 dB m^{-1}), including a constant period of Λ = 5 mm (blue), a set of different but constant periods ranging from Λ = 0.5 mm to Λ = 2 mm (yellow) and a gradually increasing period length (green). (b) Color-matched spectra of the configurations of (a), with the yellow line marking the peak value and wavelength of the $q = \pm1$ QPM peak for a range of different, but constant period lengths.

phase-matching opportunities are possible along the fiber. As the local amplification length considering a constant period is limited to one period length before Λ changes, the overall conversion efficiency drops compared to constant period lengths along the entire fiber. However, a smooth uniform creation and smearing out of the QPM peaks cannot be observed, rather individual peaks are generated that resemble the spectrum when using very long period lengths, for example Λ = 5 mm.

QPM peaks in a dual ZDW system

For all the cases investigated in Figure 4, a dispersion landscape with a single ZDW is responsible for the shape of the output. Specifically, all QPM peaks are outside the main spectrum, i.e., at shorter wavelengths than the DW_0 and at longer wavelengths than the soliton. In general, the presented fiber has two ZDWs where the second one is spectrally too far away to influence the physics, considering our laser parameters. However, changing the laser wavelength in simulations and increasing its power to compensate for the reduced nonlinear conversion efficiency due to the larger absolute GVD value at the new pump wavelength can reveal the effect of QPM on a two ZDW system. With the increased range towards the IR, silica absorption can not longer be neglected. It is accounted for here by an imaginary part of the refractive index included in Finite Element simulations of the effective mode index k = - $10^{0.001305\lambda[\mathrm{nm}]-9.1122}$ (linear approximation of data collected by Kitamura et al.[12]). Yet, individual O-H absorption bands are not included as they are dependent on the actual glass composition used. Because of the ultrashort pulses and the enhanced peak power assumed in simulations, the nonlinear frequency conversion happens within a few millimeters, making the system less prone to absorption compared to modulation instability approaches requiring substantially longer fibers. Restricting the fiber length to be close to the DW creation length, this overall allows for operation up to 4.5 µm in a silica-based system. This extended spectral range leads to an increase in mode size, demanding an enlarged field in finite element simulations to accurately model the dis-

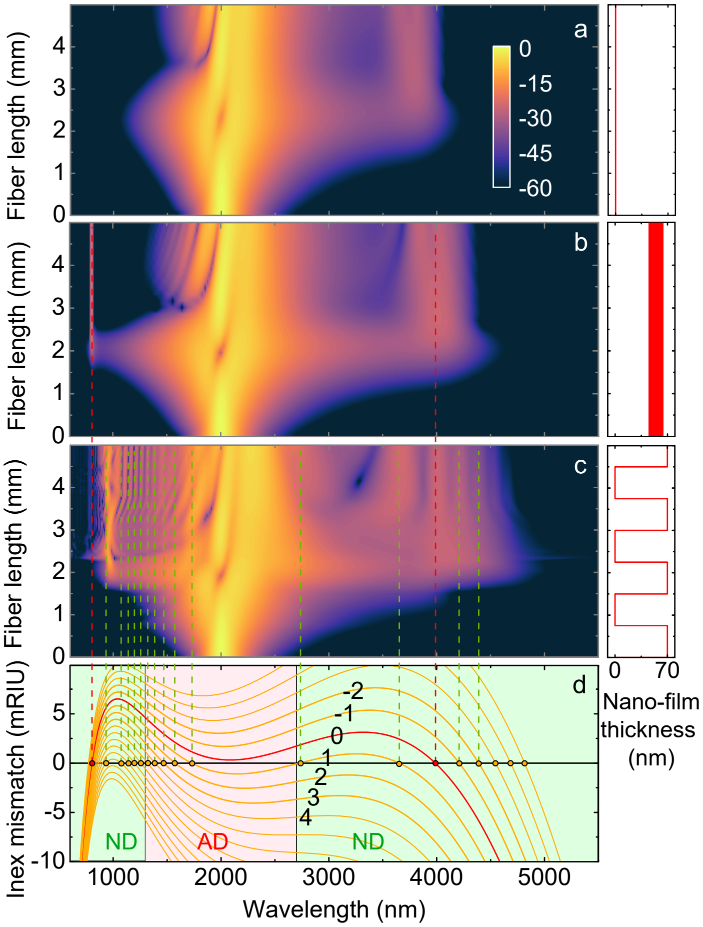

Figure S10: Ultrafast QPM-DW generation in case of two ZDW. Simulated spectral evolution of a pump pulse at λ_p = 2 µm with a peak power of 70 kW and a pulse duration of 35 fs propagating along a 5 mm long ECF without nano-film (a) and with a periodically coated 70 nm Ta_2O_5 nano-film (Λ = 1.5 mm)) considering effective dispersion (b) and alternating dispersion (c). The color coded intensity is displayed in dB. To the right are the respective nano-film thicknesses along the fiber. The distribution of the phase mismatch based on the effective dispersion and a soliton wavelength of λ_s 2.1 µm is shown in (d) with selected phase-matching wavelengths marked with dashed lines. The red curves correspond to the $q = 0$ case, highlighting the DWs possible without QPM.

persion. Therefore, the fiber core visible in the SEM image (Figure S2d) is artificially attached to three straight elongated struts with a constant profile matching the waist of the realistic, but shorter struts. Launching pulses with a central wavelength of $\lambda = 2$ µm, a pulse width of 35 fs, and a peak power of 70 kW enables exploiting both ZDWs with the creation of DWs on both sides of the spectrum (Figure S10). Although the increase in pulse energy, still only one soliton is generated ($N = 2.5$), keeping the interpretation of individual peaks straightforward and maintaining high coherence.

Without any nano-film, a soliton is generated that creates a DW only on the long wavelengths side across the second ZDW (Figure S10a). Using the effective dispersion in case of a periodically coated ECF, simulations show the creation of a short wavelength DW at $\lambda = 802$ nm isolated and leaving a gap of 700 nm to the next equally strong spectral feature (Figure S10b). The blue shifted position of the DW compared to the case presented in Figure 2 is due to the longer laser wavelength and can be confirmed by phase-matching calculations (red curve in Figure S10d). Enabling QPM by a 70 nm thick nano-film on the ECF with a period of $\Lambda = 1.5$ mm not only creates new phase-matching wavelengths outside of the classical spectrum without QPM, but also in-between the first DW and the pump (Figure S10c). The amount of QPM peaks filling up the gap increases with longer period lengths. Furthermore, QPM not only helps to create a more uniform spectrum on the short wavelength side, but also enables PM opportunities near the second, long wavelength DW_0. These additional peaks within the two DW_0s are especially numerous for dispersion landscapes with high dispersion variation, hence stronger phase mismatch. Thus, the fibers lacking a flattened dispersion profile and being less attractive for conventional nonlinear frequency conversion benefit the most from QPM enhanced PM at a fixed period length. For the short fiber lengths in this simulation with $\Lambda = 1.5$ mm, the total number of periods along the fiber is less than six with only two of them effectively contributing to the creation of the final spectrum. This helps to create QPM peaks with a broader spectral bandwidth. The central region of the spectrum, including pump and soliton is slightly different from that of the effective simulation. Although the differences are minor, a perfect match could be achieved when using a shorter period length. This can be explained by the loss of the validity of the effective dispersion, as the nonlinear length ($L_{\text{NL}} = 0.59$ mm) is reduced with the stronger peak power and, consequently, requires shorter period lengths to allow accurate prediction of the QPM-peak free spectrum. That is also why the calculated QPM wavelengths in-between the first DW_0 and the pump wavelength do not perfectly match the simulated peaks. However, the simulation can be explained qualitatively and shed light on the spectral distance of the QPM peaks. With new PM wavelengths all over the spectrum, QPM massively enhances nonlinear frequency conversion to create more uniform and broader spectra within very short fiber lengths. On the one hand, having longer fiber lengths would lead to a strong absorption of spectral features above 3.5 µm. On the other hand, this enables the Raman effect to shift the soliton to $\lambda = 2.5$ µm at a fiber position of 30 mm. With the new soliton wavelength, the PM condition changes and, for example, creates the long wavelength DW_0 near $\lambda = 3.2$ µm, at shorter wavelength but with enhanced conversion efficiency. However, the most broadband spectra are only achievable close to the DW creation length.

Lossless long wavelength potential

Artificially increasing the laser power to achieve a potential in-fiber peak power of 40 kW in simulations lead to the emergence of the second, long wavelength DW_0 near 4900 nm (Figure S11). Since the material losses of silica would prevent light generation at such long wavelengths, the losses are capped at 10 dB m^{-1} in this scenario. This allows insights and access to long wavelength features that are potentially possible to realize in fiber systems that contain materials (chalcogenides, fluoride, soft glasses, liquids etc.). In comparison to the case of Figure S10, the DW_0 is created at longer wavelength and, more interestingly, the QPM peaks enabled by a period length of 1 mm of the 70

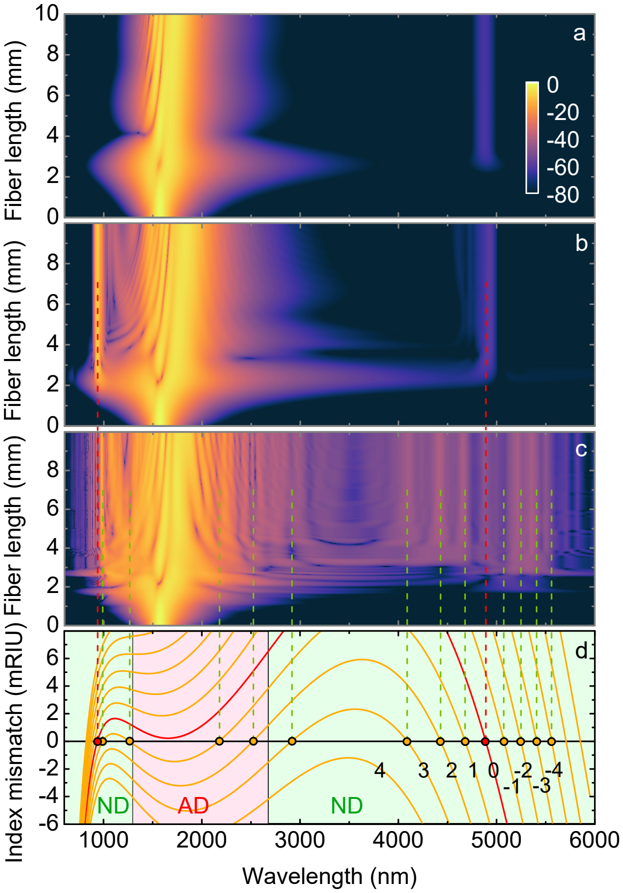

Figure S11: Ultrafast QPM at long wavelengths. Simulated spectral evolution of a pump pulse at $\lambda = 1.57$ µm with a peak power of 40 kW and a pulse duration of 35 fs and constant losses of 10 dB m^{-1}. The fiber is an uncoated ECF (a) and an ECF coated with 70 nm Ta_2O_5 ($\Lambda = 1$ mm) considering the effective dispersion (b) and the modulated dispersion (c). The color coded intensity is displayed in dB. The distribution of the phase mismatch based on the effective dispersion and a soliton wavelength of $\lambda_s = 1.665$ µm is shown in (d) with selected phase-matching wavelengths marked with dashed lines. The red curves correspond to the $q = 0$ case, highlighting the DWs possible without QPM.

nm thick nano-film are considerably (20 dB) stronger than the peak from the effective dispersion simulation. The QPM peaks on the short wavelength side of the second DW_0, but not the ones on the long wavelength side match the PM calculations, indicating that the effective dispersion is not perfectly representing the modulated case (L_{NL} = 0.9 mm < 1 mm = Λ). Nonetheless, the QPM peaks are also stronger than the DW_0 of the modulated dispersion simulation. This proves the potential of QPM conversion efficiency over regular DW generation in several cases.

References

1. Hickstein, D. D. *et al.* Quasi-phase-matched supercontinuum generation in photonic waveguides. *Phys. Rev. Lett.* **120**, 053903 (2018).

2. Lühder, T. A. K., Schneidewind, H., Schartner, E. P., Ebendorff-Heidepriem, H. & Schmidt, M. A. Longitudinally thickness-controlled nanofilms on exposed core fibres enabling spectrally flattened supercontinuum generation. *Light Adv. Manuf.* **2**, 21 (2021).

3. Droques, M., Kudlinski, A., Bouwmans, G., Martinelli, G. & Mussot, A. Experimental demonstration of modulation instability in an optical fiber with a periodic dispersion landscape. *Opt. Lett.* **37**, 4832–4834 (2012).

4. Copie, F., Kudlinski, A., Conforti, M., Martinelli, G. & Mussot, A. Modulation instability in amplitude modulated dispersion oscillating fibers. *Opt. Express* **23**, 3869–3875 (2015).

5. Conforti, M., Trillo, S., Kudlinski, A. & Mussot, A. Multiple QPM resonant radiations induced by MI in dispersion oscillating fibers. *IEEE Photonics Technol. Lett.* **28**, 740–743 (2016).

6. Conforti, M., Trillo, S., Mussot, A. & Kudlinski, A. Parametric excitation of multiple resonant radiations from localized wavepackets. *Sci. Rep.* **5**, 9433 (2015).

7. Zia, H., Lüpken, N. M., Hellwig, T., Fallnich, C. & Boller, K.-J. Supercontinuum generation in media with sign-alternated dispersion. *Laser Photonics Rev.* **14**, 2000031 (2020).

8. Driscoll, J. B. *et al.* Width-modulation of si photonic wires for quasi-phase-matching of four-wave-mixing: experimental and theoretical demonstration. *Opt. Express* **20**, 9227–9242 (2012).

9. Jankowski, M. *et al.* Ultrabroadband nonlinear optics in nanophotonic periodically poled lithium niobate waveguides. *Optica* **7**, 40–46 (2020).

10. Lefevre, Y., Vermeulen, N. & Thienpont, H. Quasi-phase-matching of four-wave-mixing-based wavelength conversion by phase-mismatch switching. *J. Lightwave Technol.* **31**, 2113–2121 (2013).

11. Langrock, C., Fejer, M. M., Hartl, I. & Fermann, M. E. Generation of octave-spanning spectra inside reverse-proton-exchanged periodically poled lithium niobate waveguides. *Opt. Lett.* **32**, 2478–2480 (2007).

12. Kitamura, R., Pilon, L. & Jonasz, M. Optical constants of silica glass from extreme ultraviolet to far infrared at near room temperature. *Appl. Opt.* **46**, 8118–8133 (2007).
